# Re-thinking residential mobility: Linking lives through time and space

**DOI:** 10.1177/0309132515575417

**Published:** 2015-03-16

**Authors:** Rory Coulter, Maarten van Ham, Allan M. Findlay

**Affiliations:** University of Cambridge, UK; Delft University of Technology, The Netherlands and University of St Andrews, UK; University of St Andrews, UK

**Keywords:** life course, linked lives, population geography, practice, relationality, residential mobility

## Abstract

While researchers are increasingly re-conceptualizing international migration, far less attention has been devoted to re-thinking short-distance residential mobility and immobility. In this paper we harness the life course approach to propose a new conceptual framework for residential mobility research. We contend that residential mobility and immobility should be re-conceptualized as *relational practices* that link lives through time and space while connecting people to structural conditions. Re-thinking and re-assessing residential mobility by exploiting new developments in longitudinal analysis will allow geographers to understand, critique and address pressing societal challenges.

## I Introduction

Mobility is a central theme of geographic scholarship. In recent decades studies have re-assessed and re-conceptualized how contemporary life is configured by the movements of people, objects, capital and information (Cresswell, 2011). This growing interest in mobility is particularly prominent within population geography (Tyner, 2013), where a burgeoning literature is re-theorizing processes of international migration through concepts such as transnationalism and diaspora (King, 2012). In this paper we argue that it is time to devote similar energy to re-thinking short-distance residential mobility and immobility.^1^ While less dramatic than international migration, for many people short-distance moves and spells of residential immobility are more common experiences that are deeply entwined with their social relations, socio-economic position and patterns of daily activities.

Contextual trends provide a powerful reason to re-think residential mobility.^2^ Population and attitudinal changes associated with the Second Demographic Transition challenge scholars to conceptualize how trends such as rising rates of solo living, the growth of ‘patchwork’ families and the popularity of ‘living apart together’ are linked to new forms of residential movement (Findlay and Wahba, 2013; Jamieson and Simpson, 2013). At the same time, the economic context is changing. Not only has the global economic crisis (GEC) impacted the housing markets within which residential mobility occurs, but it has also re-positioned the key actors involved in household moves through changes in power relations, material inequalities and intergenerational relationships (Imbroscio, 2012; Mulder, 2013). Engaging with geographic debates about knowledge and power is therefore becoming ever more critical for understanding residential mobility, as well as for re-thinking immobility as an active process that can be a desired choice or a response to restrictions and constraints (Hanson, 2005).

Grappling with the implications of contextual change requires re-invigorating and extending the life-course perspective that currently underpins residential mobility research (Clark, 2013a). The life-course perspective provides a rich framework within which to re-think residential mobility as it accommodates the growing fluidity, diversity and de-standardization of 21st-century life (Bailey, 2009). However, many of the key insights of the life-course approach have yet to be fully operationalized in residential mobility research (Coulter and van Ham, 2013). In particular, studies are only just beginning to examine the importance of linking life courses together and connecting them across long periods of time (Mulder, 2013).

The life-course perspective indicates that two types of links and connections are important when re-thinking residential mobility. First, at the micro-level, the concept of ‘linked lives’ indicates that residential moves and periods of residential stability tie people into kinship and social networks extending beyond the household unit (Elder et al., 2003). These social bonds, obligations and support exchanges play a central role in the novel forms of residential movement created by demographic and economic restructuring (Mulder, 2007). For example, many young people now move repeatedly in and out of the parental home during the protracted transition to adulthood (Sage et al., 2013).

Second, residential mobility connects the life courses of individuals to the enabling, directing and constraining influences of structural forces (Mulder and Hooimeijer, 1999). These can operate at the meso-level of the neighbourhood or locality, as for instance occurs when the actions of mortgage providers, employers, landlords and local government institutions affect the supply and demand for particular types of housing in particular locations (van Ham, 2012). Residential mobility also connects individual lives to broader processes such as national housing policies and welfare systems, technological change and long-term cultural shifts; for example those associated with the Second Demographic Transition (Lesthaeghe, 2010). Re-thinking these connections can bring a heightened sensitivity to power relations into residential mobility research.

While the life-course perspective highlights how residential mobility is configured by links and connections, insights from other fields of scholarship suggest new ways in which these can be conceptualized. First, the life-course framework can be enriched by considering the *relationality* of both linked lives and structural connections (Jones, 2014). Second, insights from the ‘new mobilities’ literature indicate that residential mobility is an active *practice* rather than a depersonalized, discrete event that carries people from dwelling A to B (Holdsworth, 2013). In this framework ‘relational’ means that ‘objects can only be understood in relation to other objects’ (Jones, 2009: 491), while practices refer to acts, interactions and performances of ‘doing’ (Holdsworth, 2013). Thus, it is by ‘doing’ residential mobility and immobility that people reveal and produce the ties linking them together and connecting them to broader structures. Using these ideas to take residential mobility research in new analytical directions can help geographers to understand, critique and address a range of contemporary social challenges.

We begin the paper by sketching how the balance of interest in residential mobility and international migration has changed over time. Next, we outline the life-course perspective and show how life-course links and connections can be re-thought at both the micro and structural levels. We then focus on each of these levels in turn, explaining how re-thinking residential mobility as a relational practice can help geographers to grapple with the challenges created by contemporary demographic and economic trends. The penultimate section uses these ideas to outline a research agenda capable of harnessing developments in longitudinal data and analytic techniques to address pressing questions. Finally, we conclude by reflecting on the broader implications of re-thinking residential mobility and immobility.

## II Migration and residential mobility

**Figure 1.**
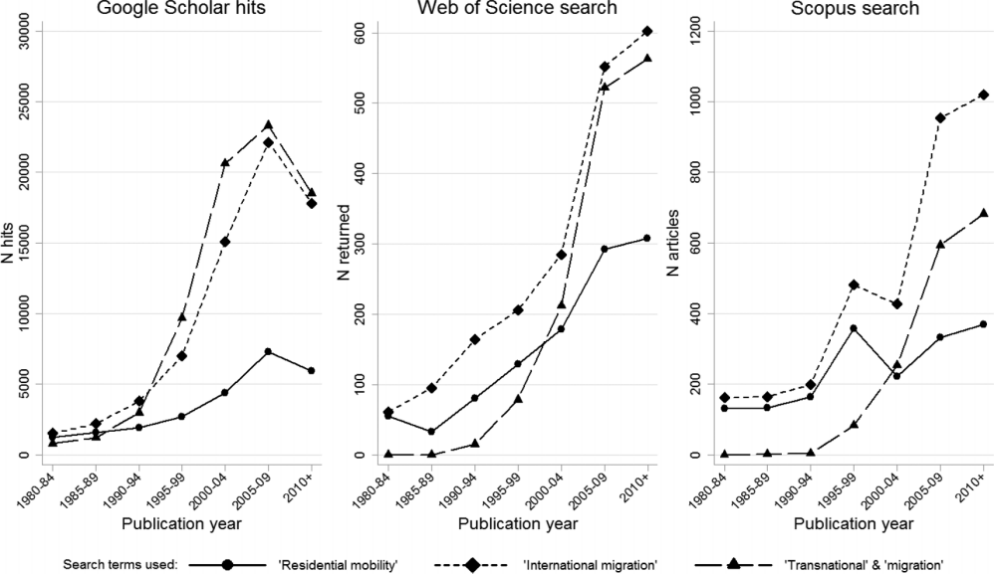
The number of documents by year of publication returned by electronic database searches for (1) ‘residential mobility’, (2) ‘international migration’, and (3) ‘transnational’ with ‘migration’. Notes: Searches were conducted on 18/04/2013. Google Scholar hits are defined as documents (excluding citations and patents) containing the search terms anywhere in the text. The Web of Science ® topic search was conducted using the Social Science Citation Index ® database. The Scopus search was conducted on the title, abstract and keywords of all articles and reviews indexed in the Scopus Social Science and Humanities database.

As scholarship on mobility proliferates and diversifies (Adey, 2009; Cresswell, 2011; Urry, 2007), population geographers have begun to call for a renewed focus on residential relocation (King, 2012). Yet given globalization and the political potency of immigration issues in many western democracies, it is unsurprising that researchers have tended to respond to this call with a renewed focus on international migration rather than short-distance residential mobility (Ellis, 2012; Tyner, 2013). With this in mind, Figure 1 presents the results of three electronic database searches exploring temporal trends in the frequency of cites to the terms ‘residential mobility’, ‘international migration’ and ‘transnational’ with ‘migration’. The figure shows the number of publications per five-year period returned by searches keyed on these terms. Each plot presents the results of searches conducted within different systems using slightly different search criteria.

Figure 1 reveals two trends in migration and residential mobility research. First, the plots show an upward trend in the number of publications referring to ‘residential mobility’ since 1980. Since the 1990s this increase has, however, been outstripped by a far more rapid rise in the number of publications focusing on ‘international migration’. All three searches suggest that, since 2010, at least two publications mentioning international migration have been produced for every one mentioning residential mobility. Although rich literatures are investigating how residential mobility is embedded in housing market conditions (Ferreira et al., 2010; Sánchez and Andrews, 2011) and implicated in neighbourhood outcomes (Hedman, 2013; Sharkey, 2012), including processes of ethnic segregation and gentrification (Hedin et al., 2012; Simpson and Finney, 2009; Smith, 2008), at the broader scale growing interest in international migration seems to be outpacing residential mobility research.

Figure 1 also demonstrates how international migration research is being enriched with new concepts. The figure shows that as migration research began to boom there was a simultaneous explosion of interest in ‘transnationalism’ (Carling, 2007; Vertovec, 2009), as well as diaspora (Cohen, 2008). Importantly, these new concepts both emphasize that migrants’ lives can only be understood through examining how their links and connections to people and places stretch across time and space (King, 2012).

## III Life course perspectives

### 1. Residential mobility and the life course

The re-theorization of international migration has not been paralleled with new perspectives on residential mobility. For over 20 years studies of long-distance internal migration and short-distance residential mobility have predominantly drawn on the life-course perspective (Clark and Dieleman, 1996; Dieleman, 2001; Mulder, 1993; Mulder and Hooimeijer, 1999). Life-course theories were first advanced in the late 20th century in response to dissatisfaction with life-cycle and generational models of human development (Elder, 1994). These approaches were perceived to be deterministic and unable to accommodate the de-standardization of life produced by contemporary changes in economic organization, education and welfare systems, family life and personal values (Dykstra and van Wissen, 1999; Elder et al., 2003). In response, the life-course perspective sought to capture this increasing dynamism and diversity by theorizing lives as trajectories made up of multiple interlinked ‘careers’, for example in the domains of employment, health and partnership (Bailey, 2009).

This sensitivity to de-standardization and diversity has greatly enriched residential mobility research (Geist and McManus, 2008). In contrast with early studies based around notions of a shared life-cycle (Leslie and Richardson, 1961; Rossi, 1955; Sabagh et al., 1969), most residential mobility researchers now regard age as a poor proxy for life-course position (Clark, 2013a). This is because the timing and ordering of life events and transitions varies from person to person (Clark and Davies Withers, 2007).

This recognition that timing matters has not, however, prompted a more fundamental re-think of conceptual models linking life-course processes to residential mobility. Most studies draw on well-established notions of ‘trigger’ events or transitions (Mulder and Hooimeijer, 1999), positing that these create disequilibrium between current and desired housing consumption (Clark and Ledwith, 2006; Kan, 1999; Littlewood and Munro, 1997; Michielin and Mulder, 2008). This then motivates an adjustment move to restore equilibrium and improve residential satisfaction (Clark et al., 2006; Diaz-Serrano and Stoyanova, 2010). In this view short-distance residential moves are often the direct result of unfolding life-course careers.

In keeping with ideas of transnationalism and diaspora, the life-course perspective stresses that individual lives are embedded within webs that stretch across space and time (Bailey, 2009). Each of Elder et al.’s (2003) five principles of life course research is underpinned by this one basic notion. Although much residential mobility research implicitly considers the importance of some types of links, these have rarely been theorized in any great detail. For example, Mulder and Hooimeijer’s (1999) ground-breaking model of mobility decision-making accommodates links and connections only in terms of micro-level resources/restrictions and structural opportunities/constraints. As we discuss in more detail in Section III.2, this approach does not completely capture the multiple ways in which life course links and connections are bound up with residential mobility.

To develop a richer conceptualization of life-course links and connections we begin by distinguishing two levels: the micro-level of *linked lives* and the meso/macro level of *structural connections* (Dieleman, 2001; Mulder and Hoomeijer, 1999). At the micro-level, Elder et al.’s (2003) principles of agency and linked lives stress that life trajectories are configured by individual choices and a person’s ties, relationships, obligations and exchanges with other people in their household, family and social networks (Dykstra and van Wissen, 1999). Although much of the residential mobility literature considers households to be the primary decision-making unit (Steele et al., 2013), the linked lives perspective implies that residential mobility and immobility are also configured by broader kinship and social geographies (Mason, 2004). These effects are fundamentally recursive as moving behaviour in turn affects social ties and interactions, thereby contributing to the consolidation, fracturing and reconstruction of families and friendship groups (Holdsworth, 2013; Smart, 2011).

Elder et al.’s (2003) principles of timing, life-span development and time and place stress the connections binding individual lives to structural conditions (Bailey, 2009). A useful starting point for conceptualizing these connections is to think of lives as biographies made up of a series of events, transitions and experiences (Dykstra and van Wissen, 1999). The timing, ordering and duration of these elements affect their meaning and implications over both the short and long term (Feijten, 2005). For example, scholars are currently debating whether fertility triggers residential moves or whether people move to particular dwellings and neighbourhoods when planning a family (Kulu, 2008; Mulder and Lauster, 2010). Studies are also beginning to examine the long-term educational, behavioural and psychological consequences of frequent residential mobility in childhood (Gasper et al., 2010; Scanlon and Devine, 2001; Tyrrell and Finney, 2014).

While the biographical metaphor conceptualizes time at the individual level (Feijten, 2005), life-course theories contend that personal biographies are further configured by the macro-contexts experienced over the life-span. The collective experiences created by these structural forces are known as period and cohort effects (Mayer, 2009). Period effects are felt by anyone living in a particular time and place, while cohort effects refer to the commonalities of experience shared by individuals who are born at the same time and live out their lives under similar structural conditions. Stockdale and Catney (2014) provide a good example of the application of these ideas in their examination of residential moves between rural and urban areas.

Although many studies relate the timing of residential moves to life events like partnership dissolution or job changes (Battu et al., 2005; Feijten and van Ham, 2010), less is known about how these relationships may vary across place, cohorts and historical time. This could be due to the changing opportunities and constraints present within housing and labour markets (Mulder and Hooimeijer, 1999). At this level, the changing actions of private and state actors such as employers, landlords, local authorities and central government can greatly alter how residential moves are linked to life-course events. For example, recent policy-driven expansion of the British higher education sector has ensured that a greater proportion of today’s young adults are experiencing the frequent residential moves associated with student life than was the case for their parents’ generation (Sage et al., 2012).

At the macro-scale, long-term changes in policies and cultural norms can also induce cohort and period effects on residential mobility. This is illustrated by Clark (2013b), who documents how the American drive for a ‘homeownership society’ has created disparities in housing wealth across birth cohorts, space and ethnic groups. Similar concerns are evident in British debates about the implications of young people’s constrained access to owner-occupation (Dorling, 2014; Heywood, 2011; McKee, 2012).

### 2 Re-thinking links and connections

Although the life-course approach provides a powerful framework within which to conceptualize and analyse residential mobility, two factors are constraining the pace of research progress. The first problem is that, until recently, data limitations meant that few researchers were able to empirically operationalize many of the key insights of the life-course perspective (Aisenbrey and Fasang, 2010). As a result, it is unsurprising that most residential mobility studies have only analysed one aspect of life courses at a time. For example, several studies have explored the timing of family events and residential moves (Michielin and Mulder, 2008), the relevance of social and kin networks (Belot and Ermisch, 2009), or the long-term associations between residential choices over the life course (Feijten et al., 2008). Fortunately, the proliferation of longitudinal data resources means that operationalizing the life-course approach is rapidly becoming much easier than ever before (Mulder, 2007).

A far more serious problem is the lack of a suitable conceptual framework to explain how residential mobility is bound up with linked lives and structural connections. Despite its great merits, the best available perspective – Mulder and Hooimeijer’s (1999) model – has several weaknesses. As noted in Section III.1, this model considers links and connections primarily in terms of tangible resources/restrictions and opportunities/constraints. However, links and connections may also be relevant for residential mobility in less tangible ways, for example if they also configure long-term goals, aspirations or desires. This indicates that it is important to think in more detail about how power relations create resources/opportunities for some people while restricting/constraining others.

Although migration scholars have developed notions of cyclical and return migration (DaVanzo, 1983; McHugh et al., 1995), most residential mobility theories focus on explaining the decision to stay or move at a single point in time (Brown and Moore, 1970; Mulder and Hooimeijer, 1999; Speare et al., 1975). This has two consequences. First, it means that linked lives and structural connections are conceptualized in terms of how they affect present behaviour, rather than in terms of how they relate to moving and staying across the life course. Furthermore, by concentrating on how links and connections affect moving decisions, existing theories overlook that residential mobility and immobility in turn affect linked lives, structural conditions and power relations. For example at the micro-level, Holdsworth (2013) argues that residential moves often affect family relations (Mason, 2004). At the broader scale, selective geographies of residential mobility and immobility are implicated in spatial processes such as gentrification, ethnic segregation, neighbourhood polarization and ‘studentification’ (Crowder et al., 2012; Hedin et al., 2012; Smith, 2008). This highlights how linked lives and structural connections are pathways through which residential mobility restructures contextual conditions and reconfigures inequalities (Sharkey, 2012).

Two ideas provide the tools for creating a richer conceptualization of linked lives and structural connections for the study of residential mobility. The first insight comes from discussions about *relationality* in economic geography (Jones, 2014; Sunley, 2008), urban studies (Jacobs, 2012) and family sociology (Mason, 2004; Smart, 2011). According to Bailey (2009), life-course perspectives are implicitly relational through time (events derive meaning from their biographical position) and space (individuals’ lives can only be understood through their links to others and their connections to structural conditions). Re-thinking residential mobility as an explicitly relational process at the level of linked lives and structural connections therefore allows us to better understand how (not) making residential moves is a process through which agents and structures interact and influence one another. This develops the implicit discussion of agent-structure relations present in analyses of neighbourhood effects and neighbourhood change (Bailey and Livingston, 2007; Hedman et al., 2011; Sharkey, 2012), as well as studies of residential mobility and social capital (Irwin et al., 2004; Kan, 2007).

The second conceptual insight comes from the outpouring of work loosely organized into what Sheller and Urry (2006) termed the ‘new mobilities paradigm’. This literature contends that movement is the defining feature of contemporary life and that various forms of mobility should be placed centre-stage in analysis (Cresswell, 2011; Urry, 2007). In essence, new mobilities scholars argue that studying movements provides a way to examine the world without privileging stability and fixed locations (Adey, 2009). This does not, however, mean that place and location no longer matter, as ‘stillness’ and ‘stuckness’ remain important experiences (Cresswell, 2012).

A key contention of this literature is that movements are active *practices* rather than discrete transitions from one location to another (Sheller and Urry, 2006). When allied to ideas of relationality, this notion provides a useful tool with which to re-conceptualise residential mobility. Re-thinking residential mobility as a relational practice rather than a discrete event can help us to understand how residential moves link lives at the micro-level by re-configuring family life and social networks (Holdsworth, 2013). At the broader scale this re-conceptualization provides a rich perspective on how the agency of individuals interacts with socio-spatial structures, as for example occurs when individuals gentrify previously working-class neighbourhoods (Smith, 2008).

Re-thinking residential mobility as a relational practice also helps us to understand immobility as an active process rather than an absence of movement (Hanson, 2005). Although most studies bundle non-movers into one group, Cresswell’s (2012) notions of stillness and stuckness highlight that residential immobility takes on different meanings depending on its duration and whether moving is desired or not (Coulter, 2013). Furthermore the spatial ‘moorings’ established through residential immobility are crucial ‘anchors’ around which people actively structure their everyday practices of mobility, for example through commuting, leisure travel and digital interactions (Cresswell, 2012; Haugen, 2012).

In summary, re-thinking residential mobility as a relational practice can enrich the life-course perspective by providing a richer conceptualization of how (not) moving is bound up with linked lives and structural connections. In the next section we examine how contextual changes – most notably those associated with the Second Demographic Transition and GEC – are creating new challenges for researchers which require re-thinking residential mobility at the levels of linked lives and structural connections. For each of these levels we discuss several ways in which re-conceptualizing residential mobility as a relational practice can advance knowledge and enable scholars to understand, critique and address current social challenges (Hamnett, 2011).

## IV Linked lives

### 1 Re-defining residential mobility

While researchers are well aware of the difficulty of defining what constitutes residential mobility and how this differs from migration (Fielding, 2012; Niedomysl, 2011), the most commonly cited definitions are rapidly becoming inadequate. Drawing on normative expectations of a linear life-cycle, traditional approaches conceptualized residential moves as discrete one-way transitions from one dwelling to another (Roseman, 1971; Rossi, 1955). When the possibility of repeated or cyclical mobility was acknowledged, this was typically considered to be a feature of long-distance migration (DaVanzo, 1983). Little attention was paid to repeated or cyclical short-distance moves or those people who regularly circulated between multiple residences (McHugh et al., 1995).

Changes in family structures and patterns of domestic living mean that continuing to define residential mobility as a discrete one-way transition overlooks an increasing proportion of residential mobility experiences. Instead of substituting one dwelling for another, these typically involve making frequent and often repeated or rhythmic moves between multiple residences (McHugh et al., 1995). This disrupts the conventional assumption that residential mobility is usually a major and unusual life event (Ferreira and Taylor, 2009).

Two overlooked forms of residential mobility have become particularly relevant in recent decades. The first is residential itinerancy. Rather like seasonal migration, we can conceptualize residential itinerancy as frequent shifts between multiple maintained residences which each function as temporary ‘centres of gravity’ around which people order their daily lives. Residential itinerancy is becoming increasingly relevant because historically high separation and divorce rates mean that contemporary western societies contain many divorcees, lone parents and children living in reconstituted or patchwork families (Beaujouan and Ní Bhrolcháin, 2011; Gram-Hansen and Bech-Danielsen, 2008). Joint custody arrangements following partnership dissolution often create residential itinerancy amongst children, for example when they live with their mother but regularly spend nights with their father. Furthermore, the popularity of ‘living apart together’ (LAT) and commuter partnerships, driven partly by rising levels of female employment, mean that many adults also divide their time between dwellings as they use residential itinerancy to juggle their life-course careers (Duncan et al., 2013; van der Klis and Mulder, 2008).

Demographic research also suggests the increasing prevalence of residential transience; particularly amongst young people leaving home, navigating higher education and entering the labour market (Sage et al., 2013). Residential transience can be defined as occurring when people move in an unstructured fashion between residences (for young adults this often involves using the parental home as a safety net), without having a single centre of gravity (Stone et al., 2011). The growth of this form of residential mobility is linked to policy shifts, for example in higher education funding and welfare provision, as well as the long-term changes in labour markets that have eroded young people’s economic position and security (Aassve et al., 2013; Berrington et al., 2009; Heath and Calvert, 2013).

Understanding residential itinerancy and transience requires considering residential moves as practices rather than transitions. This can help us to better understand why people make particular types of move at different points in the life course. While existing theories explain residential mobility as an adjustment response to housing disequilibrium or dissatisfaction (Clark et al., 2006), this model performs poorly when applied to residential itinerancy or transience. Re-thinking residential mobility as a practice overcomes this issue by guiding us to consider mobility as an adaptive strategy (Moen and Wethington, 1992), through which kinship and social bonds can be harnessed to respond to life events, pressures or structural conditions. Such practices are explicitly relational as they link life courses together; thereby restructuring families, labour markets and cultural attitudes toward domestic living arrangements.

### 2 New perspectives on mobility decision-making

Re-considering what constitutes residential mobility highlights the necessity of re-thinking how people make moving decisions. The dominant models of mobility decision-making are rooted in behaviouralism, casting moving decisions as an individualized cognitive process that is shared by all household members (Brown and Moore, 1970; Rossi, 1955; Speare et al., 1975). While these models provide a useful basis for understanding moving decisions, they have relatively little to say about the role of linked lives or unequal power relations (Halfacree and Boyle, 1993).

Re-thinking residential mobility as a relational practice helps to address these deficiencies. An important first step is to contest the notion that households are unified entities within which everyone shares the same preferences, aspirations and goals (Dieleman, 2001; Steele et al., 2013). For example, recent work demonstrates how the moving desires of both partners in couples interact to condition whether or not households relocate (Coulter et al., 2012; Ferreira and Taylor, 2009). Rabe and Taylor (2010) have extended this perspective by showing that the neighbourhood outcomes of moves are influenced by the subjective evaluations of both partners in couples.

These studies indicate how re-thinking residential mobility as a practice rather than an event can deepen our understanding of the roles that intra-household bargaining, negotiation and trade-offs play in configuring patterns of short-distance residential mobility and immobility. This is important for incorporating the sensitivity to gendered power relations present within the family migration literature into models of residential mobility decision-making (Abraham et al. 2010; Bailey et al., 2004; Cooke, 2008; Geist and McManus, 2012). This will, in turn, allow scholars to examine the extent to which gendered power structures and labour market experiences are (re)produced by residential mobility as well as long-distance migration (Halfacree, 1995)

Incorporating a deeper sensitivity to power relations requires re-considering the temporal dimension of moving decisions. While many analyses concentrate on moving events (Clark and Huang, 2003; Geist and McManus, 2008; Michielin and Mulder, 2008), a growing body of research illuminates how moving decisions are practices that unfold over time. By analysing the temporal relationships between dissatisfaction (Diaz-Serrano and Stoyanova, 2010), moving desires (Coulter et al., 2011), moving intentions (De Groot et al., 2011; Lu, 1999) and actual moving behaviour, studies are unpacking how moving decisions are bound together over time and situated within life-course biographies (Coulter and van Ham, 2013).

Importantly, this work reveals that people frequently do not behave in accordance with their previously expressed desires and intentions (De Groot, 2011). While this can be because unanticipated events disrupt decision-making (De Groot et al., 2011), the GEC highlights how structural power relations and material inequalities configure whether people are able to move or stay in accordance with their underlying preferences (Coulter, 2013). In Britain, spatially polarized house prices and rents, low rates of wage growth and reductions in social benefits have created the conditions for unwanted residential mobility (for example of social tenants in response to the ‘bedroom tax’), as well as undesired immobility (for instance due to negative equity or while saving for a mortgage deposit) (Rabe, 2012). Little is known about the long-term consequences these experiences may have for well-being or material prosperity (Nowok et al., 2013). Re-thinking residential mobility and immobility as relational practices that unfold over time can thus yield new insights about how long-term life trajectories are influenced by inequalities.

### 3 Integrating social support

Concerns about pension provision, welfare restructuring and the prevalence of casual and low-paid employment are all prompting debate about how informal support can and should be used to complement or replace state assistance (Falkingham et al., 2010). Yet by conceptualizing linked lives primarily in terms of resources and restrictions (Mulder and Hooimeijer, 1999), existing theories overlook the ways in which residential mobility can be a strategy to provide or receive social support. Re-thinking residential mobility less as a transition and more as a relational practice linking lives together can thus help us to better understand how, why and when people move in particular ways or stay in particular places in order to support or be helped by others.

Demographic trends such as population ageing, increased rates of solo living in midlife and the high prevalence of union dissolutions and lone parenthood make it crucial to understand how people use residential (im)mobility to facilitate the exchange of physical care, childcare and other forms of assistance within their social and kin networks (Bell and Rutherford, 2013; Jamieson and Simpson, 2013). Although several studies show that the residential locations of friends and family condition moving decisions and destination choices (Belot and Ermisch, 2009; Hedman, 2013; Michielin et al., 2008), few analyses consider the nature or depth of the actual contacts and exchanges between linked individuals (Blaauboer, 2011; Chan and Ermisch, 2011; Michielin et al., 2008). This is partly due to data limitations, but also because residential mobility theory concentrates on the functional ways in which linked lives act as resources or restrictions.

By focusing on residential moves rather than life-course trajectories, existing perspectives downplay how the qualitative nature of interpersonal bonds ebbs and flows over time in response to changes in the linked life courses of family members or friends (Bailey, 2009). Social and kin ties may thus only affect moving behaviour at particular flashpoints in the life course (Chan and Ermisch, 2011), for example following relationship breakdown or if health deteriorates in extreme old age (Michielin et al., 2008). Relational links may therefore be particularly important when vulnerable, living alone or when negotiating the increasingly drawn out, reversible and ‘fuzzy’ transition out of the parental home (Demey et al., 2013; Stone et al., 2011). Understanding the shifting nature of the links tying lives together requires re-thinking residential moves as unfolding practices rather than discrete events. By taking this long-term view, researchers will be better able to understand the role that residential mobility plays in the (re-)construction of families and social networks (Holdsworth, 2013).

Paying greater attention to the qualitative nature of linked lives can also bring a heightened sensitivity to power relations into analyses of residential mobility. While social capital theories construct linked lives as beneficial resources (Magdol and Bessel, 2003), demographic trends indicate that this is not always the case. High rates of partnership dissolution are creating new forms of spatially constrained mobility (Feijten and van Ham, 2013; Mulder and Malmberg, 2011), while the growth in reconstituted families means separate households are increasingly bound together through the joint custody and residential itinerancy of children (Mulder and Wagner, 2010). Although this is partly captured by the idea that linked lives can function as restrictions (Mulder and Hooimeijer, 1999), re-thinking residential mobility and immobility as active practices provides a useful way to conceptualize how a constrained ability to control one’s residential location also affects other life trajectories; such as (re)partnership options or career progression.

The importance of situating linked lives within the context of power and material inequalities has increased with recent structural changes in housing systems. These trends mean that residential mobility is becoming an increasingly relevant mechanism for the transmission of resources and inequalities over time and between birth cohorts (Clark, 2013a). As house prices in many areas have risen faster than wages while borrowing conditions have tightened (Heywood, 2011), young people are becoming increasingly dependent upon (grand)parent support when making the transition to owner-occupation (McKee, 2012). This exacerbates intragenerational inequities and reshapes intergenerational relationships (Heath and Calvert, 2013), highlighting how residential mobility and immobility can reconfigure the nature of emotional bonds as well as kinship geographies.

Residential mobility and immobility are also crucial for the (re)production of intergenerational inequalities in housing wealth. While changes in tenure patterns, housing policies (such as Right to Buy in the UK) and boom-bust cycles mean that some cohorts have accumulated more housing wealth than others (Clark, 2013b), these imbalances can be ameliorated by downsizing and direct transfers. In an era of state retreat, equity release can smooth intergenerational inequalities (for example if used to support the education or homeownership of children) or exacerbate them (if used to fund consumption or retirement) (Wood et al., 2013). This indicates that current and past practices of residential mobility and immobility are tightly bound up in the temporal reproduction of inequalities through the medium of linked lives. Unpacking these processes requires thinking of residential mobility and immobility as practices through which people use their linked lives to adapt to events and navigate structural conditions.

## V Structural connections

### 1 Re-thinking residential immobility

Re-thinking residential mobility can yield new insights about the connections between life courses and structural conditions. One way this can be achieved is by re-conceptualising residential immobility. According to Hanson (2005), residential immobility is generally conceptualised as simply an absence of movement which is not worthy of study. This is partly due to the utility-maximising framework underpinning disequilibrium and dissatisfaction based theories of decision-making (Clark et al., 2006). By focusing primarily on moving events, these perspectives tend to assume that those people who do not move are either content or actively striving to move. This overlooks those individuals who may want to move but are unable to do so (Coulter, 2013).

Contemporary trends suggest several reasons to re-conceptualize how residential immobility is embedded within structural connections. Contrary to postmodern narratives of hyper-mobility, emerging evidence suggests that rates of long-distance migration are declining in some western societies, most notably the US (Cooke, 2011, 2013; Fischer, 2002). A variety of explanations have been posited for this long-term trend. These point to structural economic shifts, increased proportions of dual-earner households, the growing ease of long-distance commuting, high rates of homeownership, an ageing population, and the impact of new communications technologies (Cooke, 2011; Green, 2004; Kaplan and Schulhofer-Wohl, 2012). At present, little is known about the relative importance of these factors or whether migration rates are declining elsewhere (Cooke, 2013). Preliminary evidence for the UK does, however, suggest that moving propensities have fallen since the 1970s, primarily due to a decline in short-distance residential mobility (Champion and Shuttleworth, 2014).

Even if we cannot generalize from these findings, evidence that some societies have falling migration and residential mobility rates does suggest a need to revisit Zelinsky’s (1971) notion of ‘mobility transitions’ (Cooke, 2011; Skeldon, 2012). This requires re-thinking residential immobility as a relational practice tying life courses into macro-level structures exerting cohort and period effects. These effects may be quite short-term, as for example occurs when residential mobility stalls during housing market busts (Ferreira et al., 2010). Declining mobility rates in times of hardship thus suggest that it is important to distinguish occasions when immobility can meaningfully be thought of as a ‘choice’ (stillness), from situations where it is the product of constraints (stuckness) (Cresswell, 2012). This requires re-thinking residential immobility as an active practice rather than as an absence of movement (Hanson, 2005).

Considering residential immobility to be a practice further draws our attention towards how long-term changes are reshaping the meaning and implications of immobility (Cooke, 2013). Cheaper transportation and new modes of instant communication not only enable commuting and teleworking (Green, 2004), they also allow social and kin relations to be stretched across space (Klinenberg, 2012; Smith and King, 2012). This suggests that the propinquity of social contacts may be becoming less of a factor in mobility decision-making than was assumed by traditional theories (Belot and Ermisch, 2009). However, if access to transportation and communications technologies remains stratified by factors such as age, gender, class, ethnicity and region, then the declining importance of propinquity may be confined to particular groups. This could create new inequalities in how immobility is experienced, highlighting the importance of incorporating power relations into analyses of structural connections.

### 2 Analysing power relations

In keeping with population geography, much writing about residential mobility is self-consciously scientific and based around sophisticated quantitative analyses of large datasets (Bailey, 2005; Clark, 2008; Findlay, 2003). While these analyses have greatly enhanced our understanding, the dearth of alternative epistemological and methodological viewpoints has created little pressure to re-theorize the life-course approach to provide a richer perspective on linked lives and structural connections. This means that re-thinking residential mobility as a relational practice will not only reinvigorate the life-course perspective but will also open up new ways to investigate neglected questions.

The political response to the GEC indicates that it is essential to examine how the social construction of residential mobility and immobility is bound up with the projection of power, with consequences for life courses and social norms (Hörschelmann, 2011). This requires disentangling the complex and contradictory ways in which moving and staying are discursively constructed. For instance, residential mobility is often advocated as a means to create flexible labour markets and social mobility (Battu et al., 2005). Yet at the same time population churn is thought to disrupt communities, particularly in deprived areas (Beatty et al., 2009; Finney and Jivraj, 2013). In spite of the paucity of empirical evidence, frequent mobility is also thought to be detrimental for family ties and children’s educational outcomes (Gasper et al., 2010). Residential mobility is thus linked to particular visions of who should be mobile, in what places, and at what times of life (Holdsworth, 2013). Such constructs tend to privilege moving to acquire or enhance housing capital, either by entering homeownership or ‘trading up’ (eg. DCLG, 2011: 1–2).

These notions affect individuals through their impact on social policies, which have been heavily restructured in the wake of the GEC to privilege certain forms of moving and staying. For example, the British government currently supports homeownership transitions through the Help to Buy scheme.^3^ Yet at the same time, caps in housing benefit and the introduction of the ‘bedroom tax’ restrict welfare payments to tenants judged to be living in excessively expensive accommodation or over-consuming space. The aim of these policies is to compel poor households and social tenants to make residential moves to dwellings that are deemed more appropriate for their life-course position. Moving and staying are thus increasingly bound up with the support and enforcement of particular spatial visions and performances of good citizenship (Lauster, 2010).

Taken together, these interventions indicate that Tyner’s (2013) call for a renewed focus on ‘surplus’ populations should be extended to emphasize how ‘mis-placed’ populations are governed and re-ordered. This requires going beyond disequilibrium-centric accounts of moving decisions to examine how practices of residential mobility are configured by power relations and material inequalities (for example between owners and renters) through the medium of social constructs (Imbroscio, 2012). This will not only provide a richer understanding of how structural connections influence life-course trajectories, but may also suggest new ways to enhance social justice by disrupting and contesting the exercise of power.

## VI New directions

### 1 Longitudinal approaches

Longitudinal analysis is integral to the life-course perspective and has long been advocated as a way to study the links between demographic processes, residential mobility and spatial structures (Buck, 2000; Davies and Pickles, 1985; Davies Withers, 1998). Yet although longitudinal methods have become the gold-standard approach, they have often been used in a way that does not fully capture the insights of the life-course perspective (Coulter and van Ham, 2013). Recent developments in data and methods mean that we are now poised to overcome several weaknesses of existing approaches.

The first problem is that most studies only use short ‘snap-shots’ of longitudinal data. This is most evident in studies of mobility decision-making (De Groot et al., 2011; Lu, 1999), neighbourhood transitions (Clark et al., 2014; Rabe and Taylor, 2010) and analyses linking life events to residential mobility and immobility (Geist and McManus, 2008). While valuable, these transition-based approaches can tell us little about how events and experiences are relationally situated within longer-term trajectories or structural cohort and period effects (Aisenbrey and Fasang, 2010; Stovel and Bolan, 2004).

Although these limitations are being partially addressed by event history modelling of anticipatory and lagged effects (Feijten, 2005; Kulu, 2008; Michielin and Mulder, 2008), until recently a dearth of long-term longitudinal data prevented analysts from also testing trajectory-based analytic techniques. This restriction is now evaporating. Many nationally representative panel surveys have now been running for many years,^4^ while geo-referenced census records are increasingly being linked over time and matched to health and administrative data (for example in the UK’s Longitudinal Studies). In addition, continuously updated population registers in countries such as Denmark, the Netherlands and Sweden provide long periods of rich longitudinal data about entire populations. When combined with developments in sequence analysis and multilevel modelling (Aisenbrey and Fasang, 2010; Pollock, 2007), these resources are greatly increasing the ease of analysing how residential mobility is situated within life-course trajectories and spatial structures (van Ham et al., 2014).

By gathering data prospectively, these resources enable researchers to overcome the difficulties associated with using retrospective data.^5^ Even setting aside thorny issues of recall bias and contextual conditioning (Schwartz, 2012), questions linking the subjective dimension of biographies to mobility practices simply cannot be answered using retrospective techniques. This is because it is very difficult for people to report the opinions, feelings or attitudes that they held in the past. Moreover, gathering retrospective data inevitably means that the past is to some extent filtered, interpreted and narrated through the lens of the present by both respondents and analysts (Taris, 2000). This creates a danger of producing Whiggish biographies downplaying uncertainty, inconsistency, dead-ends and negative experiences.

Perhaps the most daunting constraint facing researchers is the lack of longitudinal data about the links between individuals. Administrative and census resources rarely capture data on networks extending beyond the household unit. Furthermore, these datasets, as well as many surveys, often only allow people to report living in one place at once. This means that they are poorly suited to exploring practices of residential itinerancy and transience.

Overcoming these issues is becoming easier as new data emerge. Surveys such as the Gender and Generations Survey (Vikat et al., 2007), United Kingdom Household Longitudinal Study (Buck and McFall, 2012), Netherlands Kinship Panel Survey (Mulder, 2007) and German Pairfam project (Huinink et al., 2011) all contain rich data on a range of extra-household links, such as LAT relationships or exchanges of care and financial support. Furthermore, population registers allow individuals to be linked to their kin (Hedman, 2013), although little is known about the frequency and depth of actual contact and exchanges. This limitation highlights how innovative ‘family-centric’ data collection strategies, such as those used by Pairfam, may be particularly useful for better understanding how residential mobility links lives together. Extending this approach by using online surveys to gather data on temporary and hidden *de facto* living arrangements as well as *de jure* residential status could also help capture frequent, cyclical and short-term residential movements (Sage et al., 2013).

### 2 New questions

These empirical advances enable researchers to answer several sets of questions that emerge as a result of re-thinking residential mobility. The first concerns how the de-standardization of life trajectories combines with cohort and period effects to restructure how people practise and experience residential mobility and immobility in different times and places. For instance, contextual changes mean that events such as leaving home, parenthood and entering owner-occupation are experienced at increasingly diverse times which are strongly dependent on material resources (Berrington et al., 2009; Stone et al., 2011). Unpacking how the links between these events and residential mobility has changed over time can therefore shed light on the emergence of mobility transitions and new forms of inequality (Cooke, 2011). Incorporating spatial heterogeneity into this project requires internationally comparative analyses of the kind common in demographic and housing research (Dewilde and Stier, 2014; Lersch and Vidal, 2014), perhaps drawing on harmonized panel surveys (Frick et al., 2007).

Another series of questions centre on the long-term relationships between residential mobility and life-course careers. The challenge here is to use prospective rather than retrospective data to analyse how mobility practices are linked together into long-term trajectories (Coulter and van Ham, 2013). Developments in sequence analysis are making this an increasingly realistic objective. Sequence analyses use algorithms to compute measures of similarity between life courses which can then be grouped, visualized and explored (Aisenbrey and Fasang, 2010; Fasang and Raab, 2014). Although there is a pressing need to move beyond describing biographies (Wu, 2000), multilevel modelling and sequence analysis both provide geographers with a powerful way to link residential mobility biographies to new spatial patterns of living and working across neighbourhoods, cohorts, periods and places.

A final set of questions concern how residential mobility can produce ripple and shadow effects stretching across linked lives. Ripple effects refer to the ways in which changes in one person’s life course may have effects upon the lives of those they are linked to. Examining ripple effects requires shifting analyses from the individual level to focus on dyadic relations. This could, for example, involve examining how returns to the parental home have consequences for the well-being of both children and parents. Given concerns about pensions provision and the difficulty of accessing homeownership (McKee, 2012), researchers could also explore how equity release and financial transfers alter the intergenerational balance of wealth (Wood et al., 2013).

In contrast, shadow effects refer to the ways in which events and experiences can have long-lasting effects on a person’s life course (Elder, 1994). Shadow effects can condition economic resources, for example if relationship breakdowns or parental wealth configure residential mobility pathways and thus housing tenure attainments (Feijten and van Ham, 2010; Öst, 2012). Cultural shadows are also possible, for instance if experiencing frequent mobility in childhood or when attending higher education creates particular attitudes towards home and work that inform later decisions (Fielding, 1992; Stone et al., 2011; Tyrrell and Finney, 2014). Although these examples offer just a snapshot of promising new directions, taken together they highlight how harnessing new longitudinal data and methods can help us to better understand how residential mobility and immobility are relational practices that link lives together and connect people to structural forces.

## VII Conclusions

This paper has argued that geographers need to re-think residential mobility. Although the life course perspective has long provided a rich overarching framework, little attention has been directed toward conceptualizing how mobility is bound up with linked lives and structural connections. This makes it difficult to understand contemporary trends. Viewing moves as discrete transitions between dwellings overlooks the relational residential itinerancy and transience being generated by demographic shifts, while neglecting how the diverse meanings of residential immobility are being shaped by economic and technological change. Similarly, conceptualizing residential mobility as an adjustment response to disequilibrium can tell us little about how moving and staying are influenced by the increasingly uneven distribution of resources and power within families, localities and countries (Imbroscio, 2012). Furthermore, thinking of linked lives and structural connections primarily in terms of resources/restrictions and opportunities/constraints overlooks how residential moves can reconfigure structures; ranging from families and neighbourhoods to housing markets and cultural norms (Holdsworth, 2013; Mulder and Hooimeijer, 1999).

These issues can be overcome by re-thinking residential mobility and immobility as relational practices that link lives together and connect people to structural conditions through time and space. This suggests a range of new research questions that can be answered by harnessing the ‘new opportunities for creative research’ produced by novel longitudinal datasets and emerging longitudinal methods (Bailey, 2005: 117). Taken together, these conceptual and empirical innovations challenge residential mobility researchers to engage more deeply with life-course theories by taking their quantitative expertise in new directions. This is essential if geographers are to critique and help address pressing societal challenges such as adapting to demographic change and tackling inequality.

Developing a new residential mobility research agenda will also benefit scholarship. By enriching the way in which we conceptualize how demographic processes are bound up with linked lives and structural connections, re-thinking residential mobility provides a way to comprehensively integrate time and space into life-course theory and analysis (Bailey, 2009). This approach may be useful for sociology and other fields of population geography such as health research or studies of long-distance migration. Moreover, by using rigorous longitudinal analyses to develop a relational perspective that is sensitive to power structures and temporal dynamism, a revived residential mobility research tradition can inform other disciplinary subfields currently struggling with how to empirically operationalize ideas of relationality (Jones, 2014). Re-thinking and re-examining residential mobility and immobility thus points geographical scholarship in a host of exciting new directions.

## Notes

1. We define residential mobility loosely and subjectively as short-distance moves which tend not to ‘totally displace’ people’s daily activity spaces (Roseman, 1971). Residential mobility is often contrasted with migration, which is typically conceptualized as longer distance moves that do disrupt daily activity spaces (Niedomysl, 2011). It is, however, important to recognize that there is no simple way to distinguish migration from residential mobility. Studies using definitions based upon distance thresholds, administrative geographies and self-reported reasons for moving all show that there is no clear-cut distinction between these processes (Clark and Maas, 2013; Coulter and Scott, 2014; Niedomysl, 2011). The ‘fuzziness’ of the migration-residential mobility distinction means that many of our arguments therefore apply to processes of long- as well as short-distance mobility. We concentrate on the latter as most residential moves are made over comparatively short distances (Bailey and Livingston, 2007; Clark and Maas, 2013).

2. For the purposes of this article we focus specifically on residential mobility within the developed western societies of Europe, North America and the Antipodes. Although a detailed discussion of residential mobility in non-western countries is beyond the scope of this paper, our conceptual framework can easily be applied to a range of contextual conditions.

3. The Help to Buy scheme assists people to buy dwellings by providing government-backed equity loans and mortgage guarantees (HM Treasury, 2013).

4. Important examples include the US Panel Study of Income Dynamics (1968–), the German Socio-Economic Panel (1984–), the British Household Panel Survey/Understanding Society (1991–), the Swiss Household Panel (1999–) and the Household, Income and Labour Dynamics in Australia survey (2001–).

5. Prospective longitudinal data are collected by repeatedly gathering data from people as they move through time. For example, a prospective study would generate residential biographies by repeatedly interviewing a panel of respondents about their current housing conditions. In contrast, retrospective data are gathered about past attributes at a single point in time. A retrospective study would therefore obtain residential biographies by asking people to recount their residential histories.
